# Effect of omega-3 long-chain polyunsaturated fatty acid supplementation on heart rate: a meta-analysis of randomized controlled trials

**DOI:** 10.1038/s41430-017-0052-3

**Published:** 2017-12-28

**Authors:** Khemayanto Hidayat, Jing Yang, Zheng Zhang, Guo-Chong Chen, Li-Qiang Qin, Manfred Eggersdorfer, Weiguo Zhang

**Affiliations:** 1 Department of Nutrition and Food Hygiene, School of Public Health, Soochow University, Suzhou, 215123 China; 2 DSM Nutritional Products, Human Nutrition and Health, 4303 Kaiseraugst, Switzerland; 3 DSM Nutritional Products, Human Nutrition and Health, Beijing, 100020 China

## Abstract

**Background:**

Elevated resting heart rate (HR) has emerged as a new risk factor for all-cause and cardiovascular mortality. The effect of marine-derived omega-3 long-chain polyunsaturated fatty acid (n−3 LCPUFAs) supplementation on HR was investigated as an outcome in many clinical trials. The present study was to provide an updated meta-analysis on the HR-slowing effect of n−3 LCPUFAs, and to differentiate the chronotropic effect between eicosapentaenoic acid (EPA) and docosahexaenoic acid (DHA).

**Methods:**

PubMed and Cochrane databases were searched for relevant articles examining the effects of n−3 PUFAs on HR through May 2017. A random-effects model was used to generate the pooled effect sizes and 95% confidence intervals (CIs). The pooled effect sizes were presented as weighted mean differences (WMDs).

**Results:**

A total of 51 randomized controlled trials (RCTs) with approximately 3000 participants were included in this meta-analysis. Compared to placebo, n−3 PUFA supplementation mildly but significantly reduced HR (−2.23 bpm; 95% CI: −3.07, −1.40 bpm). Moderate evidence of heterogeneity was observed among included trials (*I* ^2^ = 49.1%, *P* heterogeneity < 0.001). When DHA and EPA were separately administered, modest HR reduction was observed in trials that supplemented with DHA (−2.47 bpm; 95% CI: −3.47, −1.46 bpm), but not in trials with EPA.

**Conclusions:**

The present meta-analysis provides strong clinical evidence demonstrating the effect of heart rate reduction by n−3 LCPUFA supplementation. When DHA or EPA administered alone, heart rate was slowed by DHA rather than by EPA.

## Introduction

The potential cardioprotective effects of marine-derived omega-3 long-chain polyunsaturated fatty acids (n−3 LCPUFAs) and fish intake have been investigated in numerous studies [1, 2]. Findings from a prospective study of male physicians without a history of pre-existing cardiovascular disease suggest that those who consumed fish at least once per week had a lower risk of sudden cardiac death (SCD) [3]. Moreover, baseline blood levels of n−3 LCPUFAs were also inversely associated with SCD in this population [4]. Similarly, n−3 LCPUFAs from fatty fish consumption or fish oil supplementation have been shown to lower the risk of SCD in several secondary prevention studies [4–7]. Although the exact physiologic mechanisms underlying this preventive effect of n−3 LCPUFAs on SCD remain unclear, it has been suggested that n−3 LCPUFAs may exert its protective effect on SCD by reducing heart rate (HR) [8]. Elevated resting HR is a potential risk factor for cardiovascular morbidity and mortality [9–12], particularly SCD. Therefore, any agent with HR-reducing effect and relatively no side effect may serve as a valuable candidate in SCD prevention. To this end, the effect of n−3 LCPUFA supplementation on HR has been investigated in a large number of randomized controlled trials (RCTs) [13–63], with most of the RCTs showing HR reduction compared to placebo. In 2005, Mozaffarian et al. [64] published a meta-analysis including 30 RCTs and reported that n−3 PUFA supplementation reduced HR by 1.6 beats per minute (bpm). Although 21 additional RCTs [43–63] have been published since then, no updated meta-analysis has been performed. Furthermore, several RCTs have separately investigated the effects of two major n−3 LCPUFAs, namely eicosapentaenoic acid (EPA) and docosahexaenoic acid (DHA), on HR [30, 31, 33–36, 40, 45, 54, 56]. However, whether EPA or DHA similarly results in HR reduction has not been systematically analyzed.

In order to provide updated evidence on the effect of n−3 LCPUFAs on HR reduction and to differentiate the chronotropic effect between EPA and DHA when they were separately administered, we carried out this meta-analysis and systematic review.

## Methods

### Search strategy

This present meta-analysis was planned, conducted, and reported in accordance with the preferred reporting items for systematic reviews and meta-analyses guidelines (PRISMA) [65]. PubMed and Cochrane databases were searched for relevant articles examining the effects of n−3 LCPUFAs on HR through May 2017. The following search terms were employed to identify relevant articles in the databases: (omega 3 fatty acids OR omega 3 OR polyunsaturated fatty acids OR PUFA OR fish oil OR marine oil OR eicosapentaenoic acid OR EPA OR docosahexaenoic acid OR DHA) AND (heart rate OR HR OR pulse). The search strategy had no restriction on language, publication date, or article type. The reference lists of the previous meta-analyses were reviewed to complement the database searches. Additionally, we also attempt to contact the authors of the original studies for unreported HR data.

### Selection and inclusion of RCTs

The studies eligible for inclusion in this meta-analysis had to meet the following inclusion criteria: (1) RCTs lasted at least 2 weeks; (2) one or more intervention groups received n−3 LCPUFA supplementation (i.e., fish oil (EPA plus DHA), purified EPA, purified DHA) or food containing n−3 LCPUFAs (i.e., fatty fish) and being compared with placebo; (3) trials reported effects on HR; and (4) the mean age of participants was ≥18 years.

### Data extraction and assessment of bias

Using a standardized data collection form, the following study characteristics were abstracted from each study: (1) first author’s last name, year of publication; (2) participant characteristics including the mean age, sex, and health status; (3) trial characteristics including the trial design, number of participants in intervention or control groups, total dose of EPA plus DHA, ratio of EPA to DHA, trial duration, type of control, dropout rate, and blinding; and (4) methods of HR assessment. Cochrane tool for assessing the risk of bias was used to evaluate the risk of bias among the included studies (Supplementary Table S1) [66]. Two authors (KH and JY) independently performed the database search, data extraction, and quality assessment. Any discrepancies regarding inclusion were resolved by group discussion.

### Statistical analysis

Omega-3 PUFA was considered as the intervention arm in this meta-analysis. If the multi-arm interventions included multiple doses of n−3 LCPUFAs, we included those with the highest dose in the meta-analysis. The mean changes of HR in both intervention and control groups were reported as differences between mean values at baseline and final. The standard deviations (SDs) for changes from baseline in each group were obtained from each trial. If not reported, the standard errors (SEs), confidence intervals (CIs), and *P*-values were all converted to SDs using a standard formula [66]. If only SDs for the baseline and final values were provided, we computed SDs for net changes using the method proposed by Follmann et al. [67] in which a correlation coefficient of 0.5 was assumed. The degree of heterogeneity across trials was assessed using *Q* and *I* ^2^ statistics. For the *Q* statistic, *P* < 0.1 was considered statistically significant; and for the *I* ^2^ statistic, the following conventional cut-off points were used: <25% (low heterogeneity), 25–75% (moderate heterogeneity), and >75% (severe heterogeneity). Potential publication bias was assessed using both Begg’s rank correlation test and Egger’s linear regression [68]. If the publication bias was detected, the trim and fill method was performed to correct the bias [69]. A random-effects model was used to generate the pooled effect sizes and 95% CIs [70]. The pooled effect sizes were presented as weighted mean differences (WMDs). To explore the possible influences of trial and participant characteristics on the pooled effect sizes, predefined subgroup and meta-regression analyses were performed according to the trial design (parallel vs. crossover), mean age of participants (<55 vs. ≥55), health status of participants (generally healthy vs. chronic condition), baseline HR (<69 vs. ≥69), total dose of EPA plus DHA (<3.5 vs. ≥3.5 g/day), individual n−3 PUFA supplementation (EPA vs. DHA), EPA to DHA ratio (<1.5 vs. >1.5), methods of HR measurement (single vs. multiple average vs. ambulatory/continuous), and type of control (olive oil vs. other). In addition, sensitivity analyses were performed to investigate the influence of a single trial on the overall effect estimated by omitting one trial in each turn. All analyses were performed using STATA version 11.0 (StataCorp, College Station, TX, USA). A *P*-value < 0.05 was considered to be statistically significant, unless otherwise specified.

## Results

### Trial characteristics

**Fig. 1:**
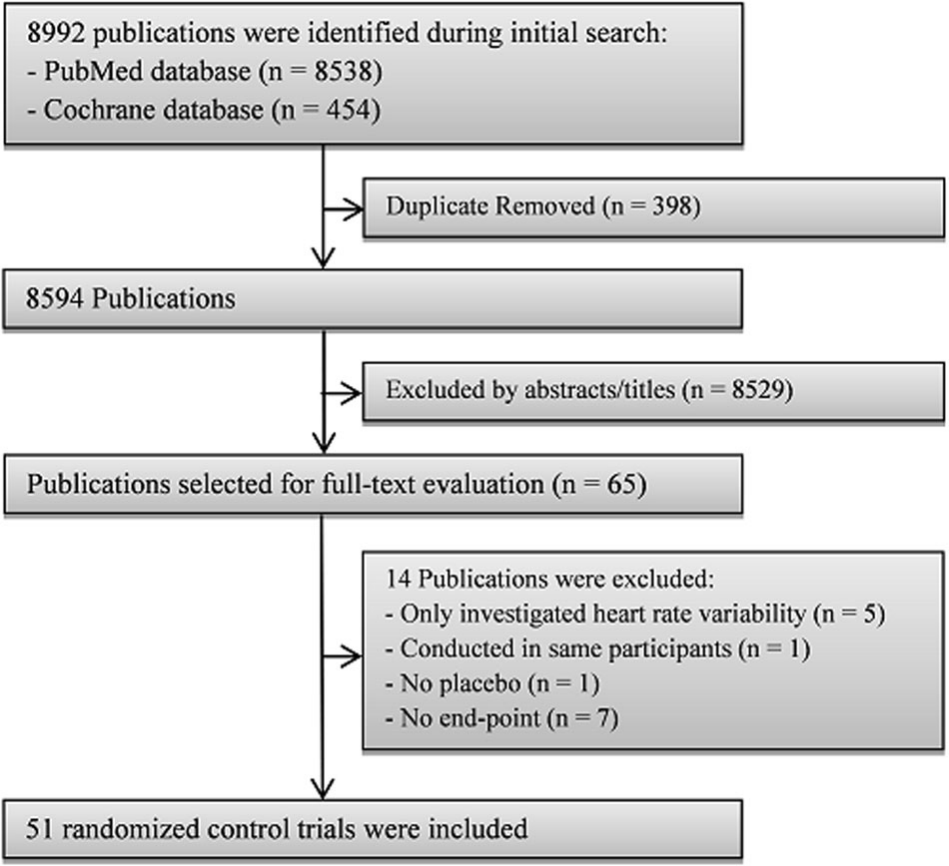
Flowchart of study selection

A flowchart of study selection, including reasons for exclusion, is presented in Fig. 1. Totally, 51 RCTs were eligible for this meta-analysis, in which 4 trials had two separated intervention groups and approximately 3000 subjects participated. The trials were published between 1988 and 2016. In term of the trial classification, 11 trials were crossover designed and 40 parallel designed; 3 trials were with single-blind treatment, 2 open-labeled, and the remaining double blind; 24 intervention groups were conducted in healthy participants, whereas the other 32 in participants with at least one chronic condition, such as coronary artery disease, renal failure, hypertension, hyperlipidemia, type 2 diabetes mellitus, frequent premature ventricular contraction, epilepsy, psoriatic arthritis, severely accident injured, and age-related cognitive decline; 19 trials were conducted exclusively in men, 2 in women, and the remaining included both sexes; the duration of trials ranged from 2 weeks to 1 year; the mean age of participants ranged from 22.45 to 70 years; HR of the participants in almost all trials was within the normal range (i.e., 60–100 bpm). The dose of EPA plus DHA ranged from 0.5 to 15 g/d, with the ratio of EPA to DHA from 0.1 to 4.9. Ten studies separately examined the differential effects of EPA and DHA. Placebo was chosen from corn oil, olive oil, safflower oil, soybean oil, sunflower oil, or mixed oil for control groups. The single HR measurement was used in 18, the average of multiple HR measurement in 23, and the average of ambulatory or continuous monitoring in 25 intervention trials. Most of the trials reported no significant side effect of LCPUFA.

### Effect of n−3 PUFAs on HR

**Fig. 2:**
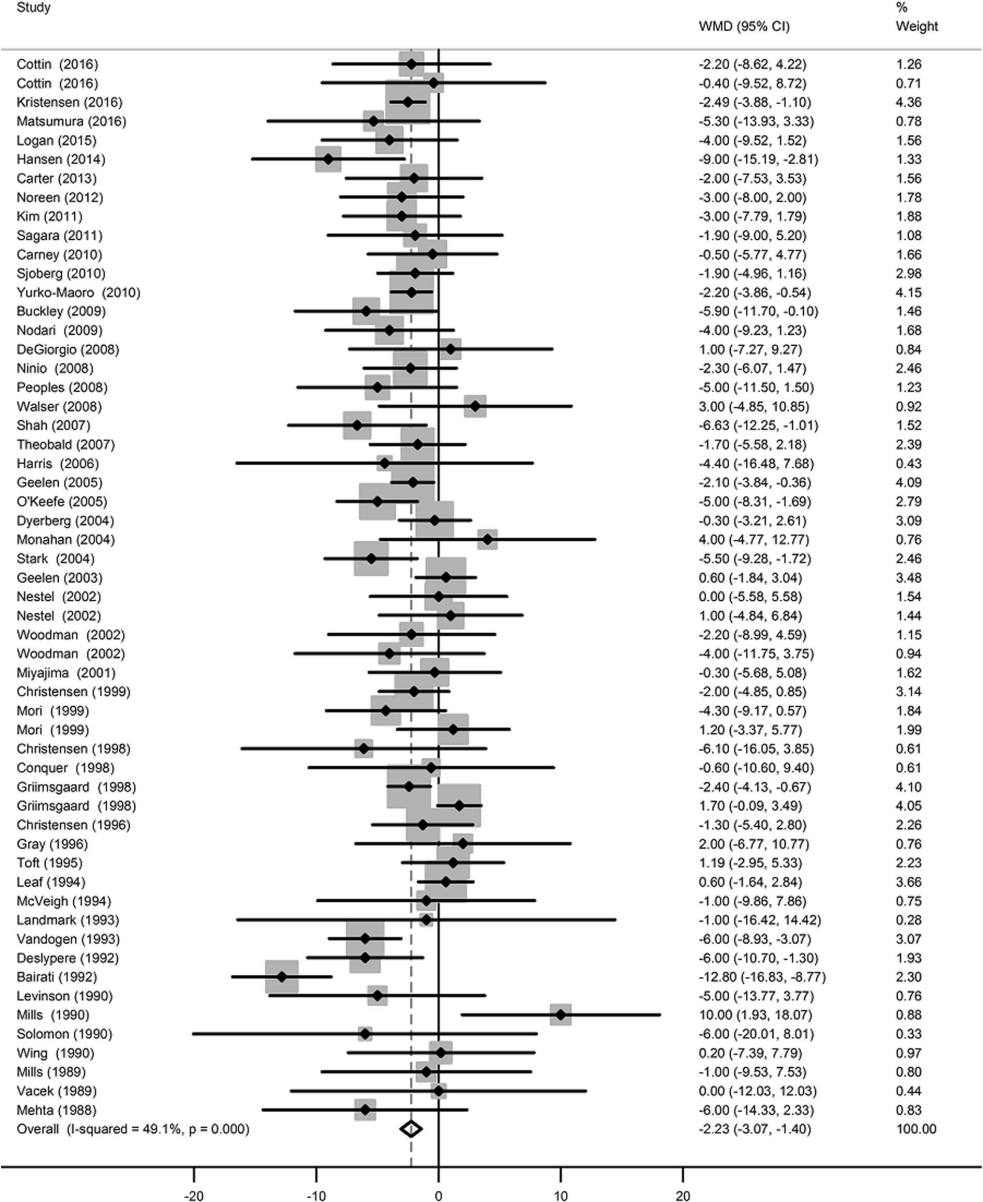
Forest plot of the change in heart rate resulting from n−3 PUFA supplementation

The net changes in HR between the intervention and control groups ranged from 0 to 10 bpm. Compared to placebo, n−3 LCPUFA supplementation mildly but significantly reduced HR (−2.23 bpm; 95% CI: −3.07, −1.40 bpm; Fig. 2). Moderate evidence of heterogeneity was observed among included trials (*I* ^2^ = 49.1%, *P* heterogeneity < 0.001). Neither Begg’s rank correlation nor Egger’s linear test observed the presence of publication bias (*P* Begg’s = 0.591, *P* Egger’s = 0.450; Table 1).

**Table 1:** Characteristics of the included 56 intervention groups (51 RCTs)

Reference	Design	Mean age (year)	Male (%)	Health status	No. of fish oil (*n*)	No. of control (*n*)	EPA+DHA (g/d)	EPA/DHA (ratio)	Duration (week)	Control	HR measurement	Dropout (%)	Blinding
Mehta et al. 1988 [13]	Crossover	63	100	CAD	8	…	5.5	1.4	4	Not available	Single	0	d
Mills et al. 1989 [14]	Parallel	28	100	Healthy	10	10	2.6	1.6	4	Olive	Single	0	d
Vacek et al. 1989 [15]	Crossover	54	63	CAD	6	…	9.0	1.5	6	Palm and cottonseed	Single	25	d
Levinson et al. 1990 [16]	Parallel	56	81	Hypertension	8	8	15.0	1.5	6	Palm and corn	Multiple average	6	d
Mills et al. 1990 [17]	Parallel	23	100	Healthy	10	10	1.3	1.6	4	Safflower	Multiple average	9	d
Solomon et al. 1990 [18]	Parallel	56	80	CAD	5	5	4.6	1.5	12	Olive	Single	0	d
Wing et al. 1990 [19]	Crossover	61	35	Hypertension	20	…	4.5	1.5	8	Olive	Multiple average	17	d
Bairati et al. 1992 [20]	Parallel	54	80	CAD	66	59	4.5	1.5	26	Olive	Single	39	d
Deslypere et al. 1992 [21]	Parallel	56	100	Healthy	14	14	2.9	4.9	52	Oleic	Multiple average	0	d
Landmark et al. 1993 [22]	Crossover	42	100	Hypertension with hyperlipidemia	18	…	4.6	0.6	4	Olive	Single	0	d
Vandogen et al. 1993 [23]	Parallel	46	100	Healthy	16	18	4.3	1.5	12	Olive, palm, safflower	Multiple average	13	d
Leaf et al. 1994 [24]	Parallel	63	79	CAD	201	205	6.9	1.4	12	Corn	Single	26	d
McVeigh et al. 1994 [25]	Crossover	53	80	T2DM	20	…	3.0	1.1	6	Olive	Single	0	d
Toft et al. 1995 [26]	Parallel	54	64	Hypertension	37	39	3.4	1.6	16	Corn	Single	10	d
Christensen et al. 1996 [27]	Parallel	NA	NA	CAD	26	23	4.3	1.5	12	Olive	24-h-continuous	11	d
Gray et al. 1996 [28]	Parallel	56	100	Hypertension	9	10	3.5	1.6	8	Corn	Multiple average	10	d
Christensen et al. 1998 [29]	Parallel	52	59	Renal failure	11	6	4.2	1.0	12	Olive	24-h-continuous	41	d
Conquer and Holub, 1998 [30]	Parallel	30	100	Healthy	9	10	3.0	Purified DHA	6	Omega 6	Single	5	d
Grimsgaard et al, 1998 [31]	Parallel	44	100	Healthy	72	77	3.8	Purified DHA	7	Corn	Multiple average	4	d
Grimsgaard et al. 1998 [31]	Parallel	44	100	Healthy	75	77	3.6	Purified EPA	7	Corn	Multiple average	4	d
Christensen et al. 1999 [32]	Parallel	38	58	Healthy	20	20	5.9	1	12	Olive	24-h-continuous	0	d
Mori et al. 1999 [33]	Parallel	49	100	Overweight, hyperlipidemia	17	20	3.7	Purified DHA	6	Olive	24-h-ambulatory	5	d
Mori et al. 1999 [33]	Parallel	49	100	Overweight, hyperlipidemia	19	20	3.8	Purified EPA	6	Olive	24-h-ambulatory	5	d
Miyajima et al. 2001 [34]	Crossover	45	100	Hypertension	17	…	2.7	Purified DHA	4	Linoleic	Multiple average	4	d
Nestel et al. 2002 [35]	Parallel	58	55	Hyperlipidemia	12	14	2.8	Purified DHA	7	Olive	Single	7	d
Nestel et al. 2002 [35]	Parallel	58	55	Hyperlipidemia	12	14	3.0	Purified EPA	7	Olive	Single	7	d
Woodmann et al. 2002 [36]	Parallel	61	76	T2DM	17	16	3.7	Purified DHA	6	Olive	24-h ambulatory	15	d
Woodmann et al. 2002 [36]	Parallel	61	76	T2DM	17	16	3.8	Purified EPA	6	Olive	24-h ambulatory	15	d
Geelen et al. 2003 [37]	Parallel	59	49	Healthy	39	35	1.3	1.2	12	Oleic	Multiple average	2	d
Dyerberg et al. 2004 [38]	Parallel	39	100	Healthy	24	25	3.2	1.5	8	Palmitic	24-h continuous	10	d
Monahan et al. 2004 [39]	Parallel	25	56	Healthy	9	9	5.0	1.5	4.3	Olive	Single	0	d
Stark and Holub, 2004 [40]	Crossover	57	0	Healthy	32	…	2.8	Purified DHA	4	Corn, soy	Multiple average	16	d
Geelen et al. 2005 [41]	Parallel	64	60	Frequent PVCs	41	43	1.3	1.2	14	Oleic	24-h continuous	9	d
O’Keefe et al. 2005 [42]	Crossover	68	100	CAD	18	…	0.8	0.4	16	Corn, olive	1-h continuous	44	d
Harris et al. 2006 [43]	Crossover	49	72	Cardiac transplant recepient	18	4	3.4	1.1	20 (median)	NA	Single	0	d
Shah et al. 2007 [44]	Parallel	31	65	Healthy	14	12	0.5	1.5	2	Corn oil	Single	4	s
Theobald et al. 2007 [45]	Crossover	48.65	50	Healthy	38	…	0.7	Purified DHA	12	Olive oil	Multiple average	0.05	d
DeGiorgio et al. 2008 [46]	Crossover	42.5	NA	Epilepsy	11	…	2.88	1.5	12	Soybean oil	1-h continuous	0	d
Ninio et al. 2008 [47]	Parallel	50.25	37	Overweight, mild hypertension, hyperlipidemia	13	14	1.92	0.2	12	Sunflower oil	Multiple average	14	d
Peoples et al. 2008 [48]	Parallel	25.15	100	Healthy	9	7	3.2	0.3	8	Olive oil	1-h continuous	88	d
Walser et al. 2008 [49]	Parallel	34.75	66	Healthy	12	9	5	1.5	6	Safflower oil	24-h continuous	9	d
Buckley et al. 2009 [50]	Parallel	22.45	100	Healthy	12	13	1.92	0.2	5	Sunflower oil	Multiple average	14	d
Nodari et al. 2009 [51]	Parallel	62.95	91	Idiopathic dilated cardiomyopathy	22	22	0.85–0.88	0.6	24	Olive oil	Single	0.07	d
Carney et al. 2010 [52]	Parallel	57.35	86	Depressed with CHD	36	36	2	1.2	10	Corn oil	Single	33	d
Sjoberg et al. 20109 [53]	Parallel	53	51	Overweight	17	17	1.91	0.2	12	Sunola oil	Multiple average	11	d
Yurko-Mauro et al. 2010 [54]	Parallel	70	42	Age-related cognitive decline	242	243	0.9	Purified DHA	24	Corn oil and soy oil	Single	10	d
Kim et al. 2011 [55]	Parallel	58.5	41	Hyperlipidemia	30	31	3.3	1.2	6	Simvastatin	24-h continuous	0.04	o
Sagara et al. 2011 [56]	Parallel	52.5	100	Hypertension and hypercholesterolemia	15	23	2	Purified DHA	5	Olive oil	Multiple average	32	d
Noreen et al. 2012 [57]	Parallel	35	100	Healthy	20	20	2.4	2	6	Safflower oil	Multiple average	0	d
Carter et al. 2012 [58]	Parallel	24	68	Healthy	34	33	2.7	1.4	8	Olive oil	Single	0	d
Hansen et al. 2014 [59]	Parallel	41	100	Healthy	42	43	3.6	0.5	23	Chicken, pork or beef	Single	12	o
Logan and Spriet, 2015 [60]	Parallel	66	0	Healthy	‘12	12	3	1	12	Olive oil	Multiple average	0	s
Cottin et al. 2016 [61]	Parallel	26.5	100	Healthy	15	15	3	Purified DHA	6	Olive oil	24-h continuous	0.02	s
Cottin et al. 2016 [61]	Parallel	26.5	100	Healthy	14	15	3	Purified EPA	6	Olive oil	24-h continuous	0.02	s
Kristensen et al. 2016 [62]	Parallel	62	41	Psoriatic arthritis	58	56	1.6	2	24	Olive oil	Single	21	d
Matsumura et al. 2016 [63]	Parallel	39.3	83	Severely accident injured	37	46	1.6	0.1	12	Rapeseed oil, soybean oil, olive oil, and fish oil^a^	Multiple average	25	d

*CAD* coronary artery disease, *CHD* coronary heart disease, *d* double blind, *NA* not available, *o* open label, *PVC* premature ventricular contraction, *s* single blind, *T2DM* type 2 diabetes mellitus

^a^Intentional addition of 63 mg fish oil to the placebo served to prevent both participants and even researchers from identifying

### Subgroup and sensitivity analyses

**Fig. 3:**
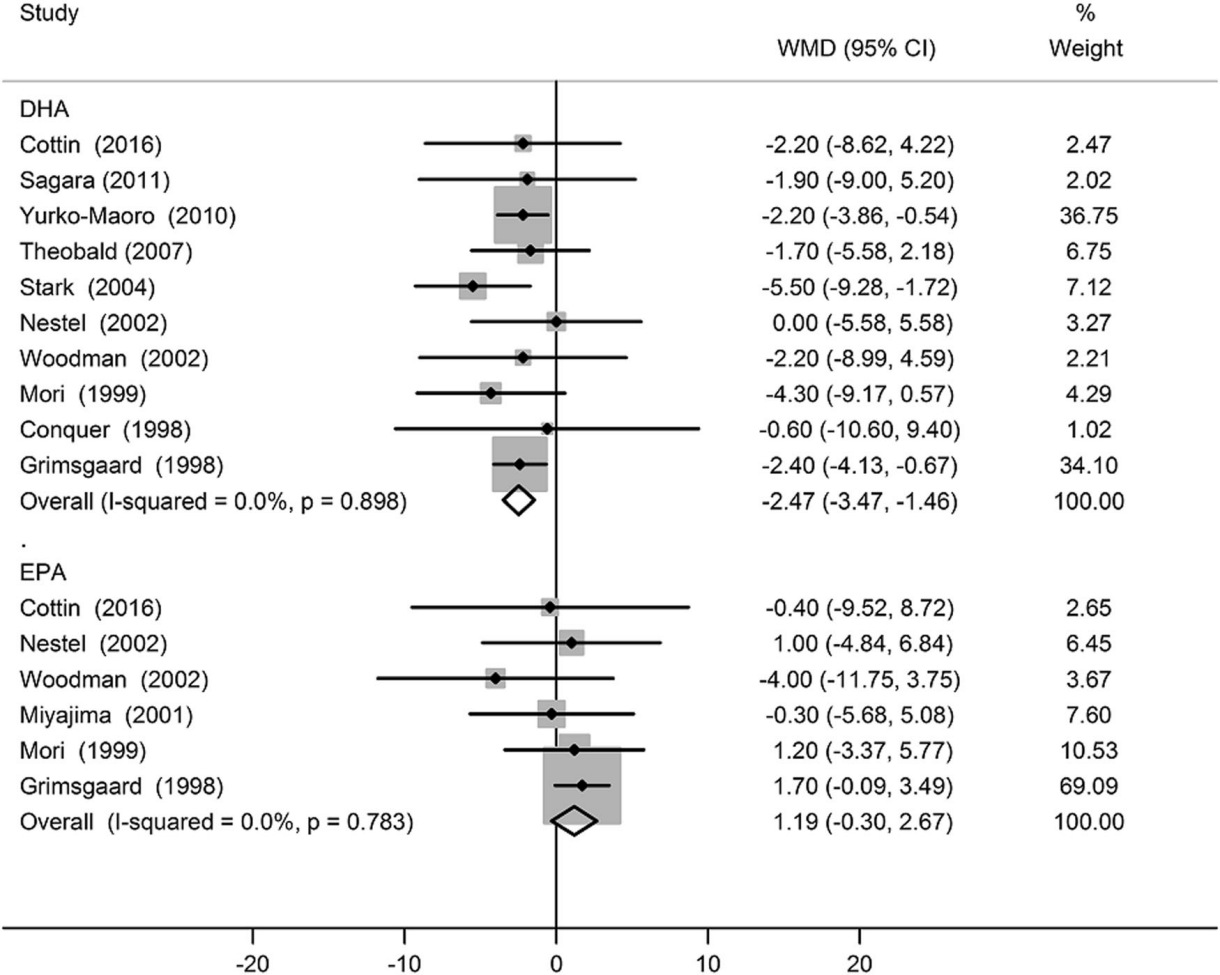
Forest plot of the change in heart rate resulting from DHA and EPA supplementations

The results of subgroup analyses according to mean age of participants, the health status of participants, baseline HR, total dose of EPA plus DHA, individual n−3 PUFA supplementation, EPA to DHA ratio, methods of HR measurement, type of control, and Jadad score are presented in Table 2. The effect of n−3 LCPUFA supplementation on HR appeared to be influenced by specific n−3 LCPUFA supplementation (*P* meta-regression < 0.01). When comparing the separate effects of EPA and DHA supplementations on HR, modest HR reduction was observed in trials that supplemented with DHA (−2.47 bpm; 95% CI: −3.47, −1.46 bpm; Fig. 3), whereas statistically significant effect was not observed with EPA supplementation (1.19 bpm; 95% CI: −0.30, 2.67 bpm; Fig. 3). The sensitivity analysis restricted to double-blind trials, and by using different values of the correlation coefficient *R* (0.25 and 0.75) did not materially alter the observed results. Moreover, the sensitivity analysis by omitting one trial in each turn revealed that the overall findings were free from the influence of a single study. In addition, the overall results remained statistically significant (−2.24 bpm; 95% CI: −3.08, −1.39 bpm) after omission of trials with duration less than 6 weeks.

**Table 2:** Subgroup analyses of the effect of n−3 PUFA supplementation on HR according to predefined study characteristics

Characteristic	Intervention groups, *n*	Effect on HR (95% CI), bpm	*P* meta-regression
Design			
Parallel	45	−2.11 (−3.06, −1.15)	0.53
Crossover	11	−2.97 (−4.30, −1.63)	
Mean age, year			
<55	35	−2.20 (−3.50, −0.90)	0.91
≥55	21	−2.12 (−3.02, −1.22)	
Health status			
Generally healthy	24	−2.21 (−3.58, −0.85)	0.93
Chronic condition	32	−2.27 (−3.31, −1.23)	
Baseline HR, bpm			
<69	26	−1.81 (−3.20, −0.42)	0.34
≥69	30	−2.50 (−3.26, −1.74)	
EPA+DHA, g/d			
<3.5	34	−2.10 (−2.82, −1.39)	0.70
≥3.5	22	−2.55 (−4.41, −0.70)	
Individual n−3 supplementation			
DHA	10	−2.47 (−3.47, −1.46)	< 0.01
EPA	6	1.19 (−0.30, 2.67)	
EPA/DHA (ratio)			
<1.5	21	−2.24 (−3.25, −1.23)	0.72
≥1.5	19	−2.19 (−4.23, −0.14)	
HR measurement			
Single	18	−2.09 (−3.70, −0.48)	
Multiple average	23	−2.03 (−3.37, −0.69)	0.52
Ambulatory/continuous	15	−1.99 (−3.01, −0.99)	
Control			
Olive oil	25	−2.60 (−3.92, −1.27)	0.37
Other	29	−1.96 (−3.06, −0.87)

### The risk of bias

The risk of bias in included studies is presented in supplementary table S1. In these trials, 17.6% presented adequate random sequence generation, 88% reported allocation concealment, 88% had blinded participants and study investigators (7 of 10), 88% had blinded assessment of outcomes, 100% had low risk of attrition bias, 84% had low risk of reporting bias, and 78% had low risk of other bias. In general, four studies were considered to be of poor quality, while the remaining studies were considered to be of fair or good quality.

## Discussion

The novel major findings from the present meta-analysis are twofold. First, by pooling the results from 51 RCTs, our study provided the latest evidence that n−3 LCPUFA supplementation reduced HR compared to placebo (−2.23 bpm; 95% CI: −3.07, −1.40 bpm); Second, by pooling results from EPA and DHA administration trials separately, our study demonstrated that DHA rather than EPA reduced HR compared to placebo (−2.47 bpm with DHA; 95% CI: −3.47, −1.46 bpm), thereby more ascribable to the negative chronotropic effect.

The effect of n−3 LCPUFA supplementation on HR was previously analyzed by Mozaffarian et al. [64] in 2005; however, it included only 30 RCTs with 1678 participants compared with 51 RCTs with approximately 3000 participants in our present meta-analysis. With more RCTs included than the previous one, this present meta-analysis provides more updated and comprehensive review of the current literature concerning the effect of n−3 LCPUFA supplementation on HR. The change in weight across trials was observed because of the significant difference in the total number of included trials. Compared to the previous meta-analysis, our findings were relatively more stable and less influenced by individual trials because a large number of additional trials were included. Moreover, our meta-analysis showed that the magnitude of HR reduction was somewhat greater than the previous meta-analysis (−2.23 bpm; 95% CI: −3.07, −1.40 bpm vs. −1.55 bpm; 95% CI: −2.51, −0.59 bpm). Furthermore, the previous meta-analysis combined multiple doses of intervention (compared to the same control) in the same meta-analysis. This approach could be problematic due to a double or triple counting of the control group, and these effect sizes from a single study might not be independent of each other. In contrast, we only included the intervention group with the highest dose to avoid this issue. Besides, several studies suggested that EPA and DHA had differential effects on HR [30, 31, 33–36, 40, 45, 54, 56], but the clarification on these issues has not been examined in the previous meta-analysis. Nonetheless, we further examine the effects of EPA and DHA supplementations on HR.

On the population level, n−3 LCPUFAs and dietary fish intake have been reported to be associated with a greater reduction in HR [71–73], which is approximated by n−3 LCPUFA supplementation that reduced HR by 2.23 bpm and DHA supplementation that reduced HR by 2.47 in this meta-analysis. It should also be further noted that the HR of the majority of participants included in this meta-analysis was within normal range—the state where reducing HR is conventionally not a medical indication [12]. At the population level however, such HR reduction may have significant public health implications, as a reduction of 3.2 bpm HR would roughly correspond to 7.5% lower risk of SCD [73]. Given the fact that both previous and present meta-analyses showed greater HR reduction trend in those with higher baseline HR, future trials may compare the effect of n−3 LCPUFA supplementation on different levels of baseline HR, particularly in those with tachycardia (resting HR > 100 bpm) or high–normal [12, 74].

The regulation of HR in humans involves multiple systems (e.g., cardiovascular, metabolic, endocrine, and autonomic nervous/neural systems) and their interactions. Aside from pharmacological drugs, lifestyle (e.g., physical fitness, psychological status, and diet or nutrition) and environment (e.g., noise and temperature) also modulates cardiac rhythm [8, 12, 64]. In vitro evidence showed that n−3 LCPUFAs directly modulated the functions of ion channels leading to reversible elevation in action potential threshold, lowering resting membrane potential and the duration of the refractory period, and finally resulting in reduction of membrane electrical excitability of cardiac myocytes [8, 12, 64]. Specifically, the inhibitory effect of n−3 LCPUFAs on funny channel current (i.e. I_*f*_), which lengthens spontaneous depolarization in cardiac pacemaker cells (i.e. sinoatrial node), is highly attributable to causing HR reduction [12, 64].

It is important to clarify the relative effects of EPA and DHA on various health outcomes in this era where n−3 LCPUFA supplements are available. The effects of individual components of n−3 LCPUFAs on HR remain poorly understood, as majority of the studies used the combination of DHA and EPA (i.e., fish oil), and more importantly, the proportion of EPA and DHA in n−3 LCPUFA supplements varied among trials. An animal study by McLennan et al. [33, 75] showing that DHA but not EPA prevented ischemia-induced cardiac arrhythmia in rats. Moreover, despite the fact that animals were given fish oil in which EPA is the dominant component, DHA appeared as the major n−3 LCPUFAs to be incorporated into myocardial membranes [76]. To date, only limited trials have examined the separate effects of DHA and EPA, with most studies showed that DHA but not EPA reduced HR [30, 31, 33–36, 40, 45, 54, 56]. Concordantly, a cross-sectional study in European men reported that DHA content of erythrocyte, but not EPA and other fatty acids, was inversely associated with HR; however, the association was slightly attenuated after further adjustment [71]. Furthermore, the inverse association between dietary fish intake and HR in observational studies [71–73] could possibly due to higher DHA content in some fish [77]. While in our general analysis, n−3 LCPUFA caused HR reduction, in our subgroup analysis, DHA but not EPA reduced HR. This may be partially explained by a greater blood pressure-lowing effect of EPA than DHA (−4.61 vs. −1.27 mm Hg in systolic blood pressure) as shown by a meta-analysis on hypertension treatment with omega-3 [78]. The pronounced blood pressure reduction by EPA can activate baroreceptor reflex [79, 80], thereby offsetting the HR-slowing effect of EPA if there is any.

This meta-analysis has several limitations that are worth mentioning. First, moderate degree of heterogeneity was observed across the included trials. Therefore, the findings from this meta-analysis should be interpreted with caution. Given that most of the trials showed a clear pattern towards the reduction of HR, the observed heterogeneity across studies was potentially due to the difference in statistical significance between trials rather than due to the difference in direction of the effect size. Second, the characteristics of participants and trials varied widely across trials, and this can lead to underestimation or overestimation of the true intervention effect. However, the predefined subgroup and sensitivity analyses showed that the characteristics of participants and trials did not affect the overall effect size. Third, the effect of individual n−3 LCPUFAs on HR was inconclusive because these findings were based on the limited evidence from RCTs. Despite these limitations, this present meta-analysis can have valuable public health and clinical implications for incorporation of n−3 LCPUFA supplementation as a lifestyle modification for reducing all-cause mortality among general populations [81], and for reducing the risk of sudden cardiac death, particularly in those who do not consume enough fatty fish on a regular basis.

## Conclusions

The present meta-analysis provides strong and updated clinical evidence demonstrating the effect of heart rate reduction by n−3 LCPUFA supplementation. In analyzing trials with DHA or EPA alone, our study demonstrates that DHA rather than EPA is more ascribable to such chronotropic effect. Future investigations may evaluate whether heart rate reduction with n−3 LCPUFAs in general and with DHA in specific is associated with improved outcomes in clinical patients or with better health profile of the public.

## Electronic supplementary material

Quality assessment of included RCTs in this meta-analysis
