# Supplementary material for: Effect of omega-3 long-chain polyunsaturated fatty acid supplementation on heart rate: a meta-analysis of randomized controlled trials
Source: Eur J Clin Nutr. 2017 Dec 28;72(6):805–17. doi: 10.1038/s41430-017-0052-3 (PMC5988646; doi:10.1038/s41430-017-0052-3)
Supplement: Supplementary file 1 — Quality assessment of included RCTs in this meta-analysis [file 41430_2017_52_MOESM1_ESM.docx]

Table S1: Quality assessment of included RCTs in this meta-analysis

| **Study (year)** | **Selection bias** | | **Performance bias** | **Detection bias** | **Attrition bias** | **Reporting bias** | **Other bias** | **Quality** |
| --- | --- | --- | --- | --- | --- | --- | --- | --- |
|  | **Random sequence generation** | **Allocation**  **concealment** | **Blinding of participants and**  **study investigators** | **Blinding of outcome assessment** | **Incomplete outcome data** | **Selective reporting** |  |  |
| Mehta et al., 1988 (13) | Unclear | Low risk | Low risk | Low risk | Low risk | Low risk | Unclear | Fair quality |
| Mills et al., 1989 (14) | Unclear | Low risk | Low risk | Low risk | Low risk | Low risk | Low risk | Fair quality |
| Vacek et al., 1989 (15) | Unclear | Low risk | Low risk | Low risk | Low risk | Low risk | Unclear | Fair quality |
| Levinson et al., 1990 (16) | Unclear | Low risk | Low risk | Low risk | Low risk | Low risk | Low risk | Fair quality |
| Mills et al., 1990 (17) | Unclear | Low risk | Low risk | Low risk | Low risk | Low risk | Low risk | Fair quality |
| Solomon et al., 1990 (18) | Unclear | Low risk | Low risk | Low risk | Low risk | Low risk | Low risk | Fair quality |
| Wing et al., 1990 (19) | Unclear | Low risk | Low risk | Low risk | Low risk | Low risk | Unclear | Fair quality |
| Bairati et al., 1992 (20) | Unclear | Low risk | Low risk | Low risk | Low risk | Low risk | Low risk | Fair quality |
| Deslypere et al., 1992 (21) | Unclear | Low risk | Low risk | Low risk | Low risk | Low risk | Low risk | Fair quality |
| Landmark et al., 1993 (22) | Unclear | Low risk | Low risk | Low risk | Low risk | Low risk | Unclear | Fair quality |
| Vandogen et al., 1993 (23) | Unclear | Low risk | Low risk | Low risk | Low risk | Low risk | Low risk | Fair quality |
| Leaf et al., 1994 (24) | Unclear | Low risk | Low risk | Low risk | Low risk | Low risk | Low risk | Fair quality |
| McVeigh et al., 1994 (25) | Unclear | Low risk | Low risk | Low risk | Low risk | Unclear | Unclear | Fair quality |
| Toft et al., 1995 (26) | Low risk | Low risk | Low risk | Low risk | Low risk | Unclear | Low risk | Fair quality |
| Christensen et al., 1996 (27) | Low risk | Low risk | Low risk | Low risk | Low risk | Unclear | Low risk | Fair quality |
| Gray et al., 1996 (28) | Unclear | Low risk | Low risk | Low risk | Low risk | Unclear | Low risk | Fair quality |
| Christensen et al., 1998 (29) | Unclear | Low risk | Low risk | Low risk | Low risk | Unclear | Low risk | Fair quality |
| Conquer and Holub, 1998 (30) | Unclear | Low risk | Low risk | Low risk | Low risk | Unclear | Low risk | Fair quality |
| Griimsgard et al, 1998 (31) | Low risk | Low risk | Low risk | Low risk | Low risk | Low risk | Low risk | Good quality |
| Christensen et al., 1999 (32) | Unclear | Low risk | Low risk | Low risk | Low risk | Unclear | Low risk | Fair quality |
| Mori et al., 1999 (33) | Unclear | Low risk | Low risk | Low risk | Low risk | Low risk | Low risk | Fair quality |
| Miyajima et al., 2001 (34) | Unclear | Low risk | Low risk | Low risk | Low risk | Low risk | Unclear | Fair quality |
| Nestel et al., 2002 (35) | Low risk | Low risk | Low risk | Low risk | Low risk | Low risk | Low risk | Good quality |
| Woodmann et al., 2002 (36) | Unclear | Low risk | Low risk | Low risk | Low risk | Low risk | Low risk | Fair quality |
| Geelen et al., 2003 (37) | Unclear | Low risk | Low risk | Low risk | Low risk | Low risk | Low risk | Fair quality |
| Dyerberg et al., 2004 (38) | Low risk | Low risk | Low risk | Low risk | Low risk | Unclear | Low risk | Fair quality |
| Monahan et al., 2004 (39) | Unclear | Low risk | Low risk | Low risk | Low risk | Low risk | Low risk | Fair quality |
| Stark and Holub, 2004 (40) | Unclear | Low risk | Low risk | Low risk | Low risk | Low risk | Unclear | Fair quality |
| Geelen et al., 2005 (41) | Unclear | Low risk | Low risk | Low risk | Low risk | Low risk | Low risk | Fair quality |
| O’Keefe et al., 2005 (42) | Unclear | Low risk | Low risk | Low risk | Low risk | Low risk | Unclear | Fair quality |
| Harris et al., 2006 (43) | Unclear | High risk | High risk | High risk | Low risk | Low risk | Unclear | Poor quality |
| Shah et al., 2007 (44) | Unclear | High risk | High risk | High risk | Low risk | Low risk | Low risk | Poor quality |
| Theobald et al., 2007 (45) | Unclear | Low risk | Low risk | Low risk | Low risk | Low risk | Unclear | Fair quality |
| DeGiorgio et al., 2008 (46) | Unclear | Low risk | Low risk | Low risk | Low risk | Low risk | Unclear | Fair quality |
| Ninio et al., 2008 (47) | Unclear | Low risk | Low risk | Low risk | Low risk | Low risk | Low risk | Fair quality |
| Peoples et al., 2008 (48) | Unclear | Low risk | Low risk | Low risk | Low risk | Low risk | Low risk | Fair quality |
| Walser et al., 2008 (49) | Unclear | Low risk | Low risk | Low risk | Low risk | Low risk | Low risk | Fair quality |
| Buckley et al., 2009 (50) | Unclear | Low risk | Low risk | Low risk | Low risk | Low risk | Low risk | Fair quality |
| Nodari et al., 2009 (51) | Unclear | Low risk | Low risk | Low risk | Low risk | Low risk | Low risk | Fair quality |
| Carney et al., 2009 (52) | Unclear | Low risk | Low risk | Low risk | Low risk | Low risk | Low risk | Fair quality |
| Sjoberg et al., 2009 (53) | Unclear | Low risk | Low risk | Low risk | Low risk | Low risk | Low risk | Fair quality |
| Yurko-Mauro et al., 2010 (54) | Low risk | Low risk | Low risk | Low risk | Low risk | Low risk | Low risk | Fair quality |
| Kim et al., 2011 (55) | Low risk | High risk | High risk | High risk | Low risk | Low risk | Low risk | Poor quality |
| Sagara et al., 2011 (56) | Low risk | Low risk | Low risk | Low risk | Low risk | Low risk | Low risk | Fair quality |
| Noreen et al., 2012 (57) | Unclear | Low risk | Low risk | Low risk | Low risk | Low risk | Low risk | Fair quality |
| Carter et al., 2012 (58) | Unclear | Low risk | Low risk | Low risk | Low risk | Low risk | Low risk | Fair quality |
| Hansen et al., 2014 (59) | Low risk | High risk | High risk | High risk | Low risk | Low risk | Low risk | Poor quality |
| Logan and Spriet., 2015 (60) | Unclear | High risk | High risk | High risk | Low risk | Low risk | Low risk | Poor quality |
| Cottin et al., 2016 (61) | Unclear | High risk | High risk | High risk | Low risk | Low risk | Low risk | Poor quality |
| Kristensen et al. 2016 (62) | Unclear | Low risk | Low risk | Low risk | Low risk | Low risk | Low risk | Fair quality |
| Matsumura et al. 2016 (63) | Unclear | Low risk | Low risk | Low risk | Low risk | Low risk | Low risk | Fair quality |
